# Nanopore sequencing of a monkeypox virus strain isolated from a pustular lesion in the Central African Republic

**DOI:** 10.1038/s41598-022-15073-1

**Published:** 2022-06-24

**Authors:** Mathias Vandenbogaert, Aurélia Kwasiborski, Ella Gonofio, Stéphane Descorps‐Declère, Benjamin Selekon, Andriniaina Andy Nkili Meyong, Rita Sem Ouilibona, Antoine Gessain, Jean-Claude Manuguerra, Valérie Caro, Emmanuel Nakoune, Nicolas Berthet

**Affiliations:** 1 Unité Environnement et Risque Infectieux, Cellule d’Intervention Biologique d’Urgence, Institut Pasteur, Paris, France; 2 Institut Pasteur de Bangui, Bangui, Central African Republic; 3 Centre of Bioinformatics, Biostatistics and Integrative Biology (C3BI), Institut Pasteur, Paris, France; 4 Centre Interdisciplinaire de Recherches Médicales de Franceville (CIRMF), Franceville, Gabon; 5 Unité d’Epidémiologie et Physiopathologie des Virus Oncogènes, Département de Virologie, UMR3569, Institut Pasteur, Centre National de la Recherche Scientifique (CNRS, Paris, France; 6 The Center for Microbes, Development and Health, CAS Key Laboratory of Molecular Virology and Immunology, Institut Pasteur of Shanghai-Chinese Academy of Sciences, Discovery and Molecular Characterization of Pathogens, No. 320 Yueyang Road, XuHui District, Shanghai, 200031 China

**Keywords:** Pox virus, Infectious-disease diagnostics

## Abstract

Monkeypox is an emerging and neglected zoonotic disease whose number of reported cases has been gradually increasing in Central Africa since 1980. This disease is caused by the monkeypox virus (MPXV), which belongs to the genus *Orthopoxvirus* in the family *Poxviridae*. Obtaining molecular data is particularly useful for establishing the relationships between the viral strains involved in outbreaks in countries affected by this disease. In this study, we evaluated the use of the MinION real-time sequencer as well as different polishing tools on MinION-sequenced genome for sequencing the MPXV genome originating from a pustular lesion in the context of an epidemic in a remote area of the Central African Republic. The reads corresponding to the MPXV genome were identified using two taxonomic classifiers, Kraken2 and Kaiju. Assembly of these reads led to a complete sequence of 196,956 bases, which is 6322 bases longer than the sequence previously obtained with Illumina sequencing from the same sample. The comparison of the two sequences showed mainly indels at the homopolymeric regions. However, the combined use of Canu with specific polishing tools such as Medaka and Homopolish was the best combination that reduced their numbers without adding mismatches. Although MinION sequencing is known to introduce a number of characteristic errors compared to Illumina sequencing, the new polishing tools allow a better-quality MinION-sequenced genome, thus to be used to help determine strain origin through phylogenetic analysis.

## Introduction

Monkeypox is an emerging and neglected disease of zoonotic origin that presents with maculopapular rashes—particularly on the palms of the hands and soles of the feet—sometimes very similar to those of smallpox^1,2^. Infection can also be associated with adenopathy. This infection is caused by the monkeypox virus (MPXV), which belongs to the genus *Orthopoxvirus* in the family *Poxviridae*. This large virus, whose genome is around 200 kb, was first isolated in 1958 from a monkey (*Macaca fascicularis*) originating from Singapore and imported to Copenhagen, Denmark that had caused an outbreak in captive *Cynomolgus* monkeys^3^. However, the precise animal reservoirs of MPXV have yet to be identified, although this virus was isolated once from a symptomatic squirrel (*Funisciurus anerythrus*) caught in 1985 in the Democratic Republic of Congo (DRC) near a village where a human case had been reported previously and once from a sooty mangabey (*Cercocebus atys*) in 2012 in Ivory Coast^4,5^. Although orthopoxvirus antibodies are not specific to MPXV, they have been detected in a large number of animal species living in Africa, including numerous non-human primates and rodents, suggesting the presence of orthopoxviruses in wild animals^6^. In Central or West Africa, outbreaks of monkeypox are generally reported in remote populations that depend on hunting and consume bushmeat^7–9^. Although the reasons are not clear, there has been a gradual increase in the number of monkeypox cases since 1980 in Central Africa (mainly in the DRC) and the Central African Republic (CAR)^10–13^), but also more recently in West Africa, particularly in Nigeria since 2017–2018^14,15^ where the previous case was reported several decades ago.Unlike the strains belonging to the West African clade, in particular that of the last epidemic in Nigeria, which have already spread in several countries outside the African continent and to Cameroon^16–18^, the strains belonging to the Congo Basin clade (Central Africa) have never been identified outside their geographical region of origin. Indeed, genomic analyses of the main MPXV strains detected in the CAR between 2001 and 2018 confirmed that they belonged to three lineages closely related to those found in DRC^19^. Finally, molecular data obtained from viral genomes can trace the origin of strains and establish relationships between strains isolated from different outbreaks in the countries affected by this disease.

The rapid identification of pathogens responsible for infectious diseases and access to high quality genome sequences are clearly essential for better disease management^20^. Molecular characterization of the pathogen is generally carried out using second-generation sequencing (SGS) techniques. However, these techniques only generate short sequences and are biased according to GC content, limiting their efficiency^21^. To overcome these limits, third-generation sequencing (TGS) techniques, including Oxford Nanopore technologies (ONT) and PacBio platforms, have been widely developed and applied to study pathogens such as bacteria and viruses^22^. Unlike PacBio, ONT sequencers, especially the MinION, are better adapted to field sequencing, because they are highly portable, being small in size and weight (< 100 g). In addition, library preparation is simpler than that used for SGS. All these technical advantages of TGS have made it possible to sequence genomes rapidly in regions with extreme climate conditions^23,24^.The most elegant demonstration of the usefulness of rapid MinION sequencing is that of the field for a rapid molecular characterization of Ebola virus during the last West African outbreak^25^. Real-time monitoring of the Ebola virus and the associated genomic analysis shed much-needed light on the spread of the virus and helped establish infection control strategies^26^. MinION sequencing has also been successfully used to study other viruses in epidemics, such as the Zika virus in South America, where MinION sequencing results helped link congenital malformations to Zika fever, or in the recent Lassa virus epidemic in Nigeria in 2019^27^. In addition to these examples, TGS has also been used to better understand the evolution of emerging pathogens in animals, and in particular to better characterize the African swine fever virus (ASFV). Although this virus is endemic to sub-Saharan Africa, China was the first Asian country to be affected by an ASFV epidemic in August 2018 and this virus has since spread to neighboring countries. The data suggest a single origin of the epidemic, but SGS and TGS have shown variation in genomes across cities and provinces in China^28^. TGS has not yet been used alone to sequence an MPXV genome; however, it has already been combined with SGS to sequence several strains, including the genome of the MPXV strain detected in a patient who discarded rodent carcasses at his home in Port Harcourt (Nigeria) before returning to Israel a week later^29,30^.

The aim of our study was to evaluate the performance and added value of the MinION real-time TGS sequencing device for sequencing the complete genome (around 200 kb) of an MPXV strain, obtained directly from a pustular lesion sampled in a remote area of Central Africa during an outbreak. At the time of writing, this study represents the first attempt to sequence using only MinION a member of the MPXV, for which there are only a small number of reference genomes available for comparison. Due to the sparsity of available reference genomes, the reads (directly obtained from the lesion) were taxonomically binned to identify the species-origin of the reads. This binning approach is similar to a metagenomics setup using two taxonomic classifiers. Assembly of the corresponding TGS reads was done using state-of-the-art assembly methods, as implemented in the Canu tool^31^. For validation, the resulting genome sequence was compared with that obtained using a different, but more conventional method (SGS). The results we obtained illustrate the usefulness of this sequencing approach to quickly obtain a whole virus genome sequence at a level of accuracy that is precise enough for phylogeographic determination of the origin of this zoonotic virus.

## Materials and methods

### Organization of suspected case notification, collection of biological samples, DNA extraction of the monkeypox virus and molecular assays

Whenever a case of monkeypox is reported in CAR, standardized data collection procedures have been developed and validated by the Ministry of Health and the World Health Organization (WHO). These procedures consist of notification of suspected cases, collection of biological samples for diagnosis and data collection in the field by the investigation team. The biological samples were then sent to the IPB in the best possible storage conditions for virological investigations. Although independent samples of several pustules were collected, DNA extraction was performed on a single pustular lesion using the QIAamp DNA Mini kit, according to the manufacturer’s instructions. Extracted DNA was quantified using a Qubit dsDNA High-Sensistivity Assay kit (Invitrogen) following the protocol according to the manufacturer’s guidelines and stored at –20 °C until use in molecular investigations. MPXV was detected using a quantitative polymerase chain reaction (PCR), as previously described ^32,33^.

### MinION library preparation and sequencing

Barcoded sequencing libraries were prepared from 400 ng DNA using the Rapid Barcoding Sequencing kit SQK-RBK004 (Oxford Nanopore Technologies) following the manufacturer’s protocol. The optional clean-up steps using AMPure XP beads (Beckman Coulter) were carried out to increase throughput. The library was loaded onto an R9.4 flow cell (FLO-MIN106) and sequenced on MinION Mk1B device within 8 h. ONT MinKNOW software (version 19.05.0) was used to collect raw sequencing data and ONT’s cloud-based basecaller based on Guppy (version 3.2.8) was used to perform on-site and real-time basecalling during sequencing runs. Subsequently, the “What is my pot?” (WIMP) workflow was launched for real-time species classification and estimation of the species (metagenomics) diversity of the sample (based on Centrifuge software).

### Bioinformatics analyses

**Figure 1:**
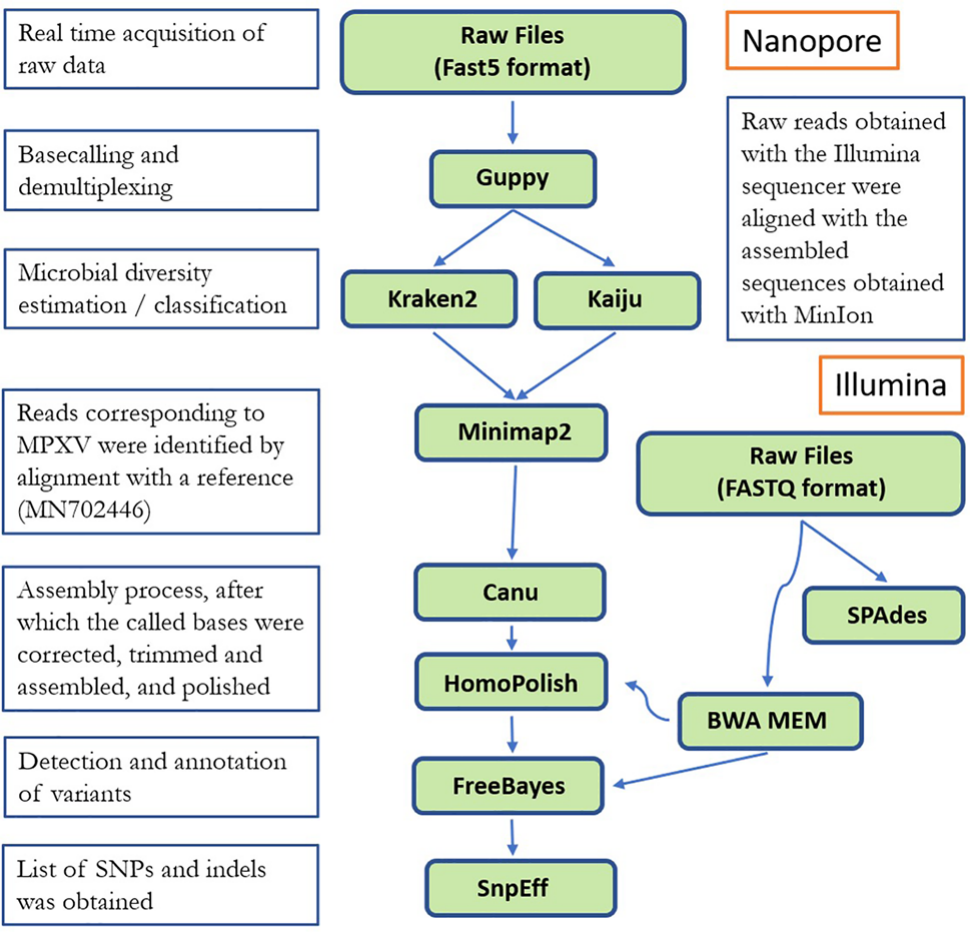
Workflow for the analysis of raw data, from real-time acquisition to the listing of SNPs and indels.

Unlike the previous steps which were carried out at the Institut Pasteur of Bangui (IPB), the following steps were carried out at the Institut Pasteur in Paris after the transfer of the raw data acquired in real time in files containing 4000 sequences in FAST5 format. These files were basecalled and demultiplexed using Guppy (version 3.4.1) (Fig. 1). Microbial diversity was determined by using (1) the Kraken2 taxonomic classifier based on a custom extended RefSeq database (containing the RefSeq reference libraries for viral, bacterial, fungal, archaea, protozoan and plasmid genomes/proteins, the human GRCh38 human genome/proteins, as well as the NCBI non-redundant nucleotide database containing sequences from large environmental sequencing projects), and (2) Kaiju, a protein-level classifier, using an equivalent protein database^34–36^ (Fig. 1). The taxonomic classification of raw reads results were visualized on the Krona web interface^37^. The identification of reads corresponding to MPXV were confirmed by alignment with a reference MPXV (NC_003310) using Minimap2 (version 2.9) for consensus sequence process, after which the called bases were corrected, trimmed, assembled and read-corrected using Canu (version 1.8)^31,38^. A caveat of long-read technology is the introduction of sequencing errors (mostly short indels), which was thus verified and corrected by polishing using HomoPolish (Release v0.3) (Fig. 1). The workflow used for the detection and annotation of variants in the MinION dataset consisted of an alignment of the reads using Minimap2 (version 2.9)^38^ to the reference sequence of the same virus (GenBank Accession MN702446) obtained by Illumina sequencing and assembled using SPAdes (version 3.10). Then, from this alignment, the list of SNPs and indels was obtained using Freebayes (version 1.1)^39^. Annotation was performed using SnpEff (version 4.3) according to the annotations of the Illumina sequence (MN702446)^40^. Finally, the raw reads obtained with the Illumina sequencer (BioProject number PRJNA680806) were compared with the assembled sequences obtained with the MinION sequencer using BWA MEM (version 0.7.4)^41,42^ (Fig. 1).

### Phylogenetic analyses

The genome of the MPXV 38c strain sequenced with the MinION was aligned with the 65 MPXV genomes available at NCBI using MAFFT v7.471 (2020/Jul/3)^43,44^. Sequences from two other poxvirus genomes (cowpox virus, Grisham 1990, X94355 and horsepox virus, Mongolia 1976, DQ792504) were used as outgroups. The resulting alignment was manually edited to remove poorly aligned portions, in particular the extremities containing missing data, as well as many insertions or deletions (indels). IQ-TREE was then used to reconstruct a global MPXV bootstrapped likelihood phylogenetic tree with 1000 replicates using the “best model” indicated in the IQ-TREE internal model fitter^45^.

### Ethical statement

The investigation of this case of monkeypox virus infection in the CAR was approved by both the Institut Pasteur of Bangui Scientific Committee and the CES (*Comité Ethique et Scientifique*; University of Bangui). All the experiments carried were done in accordance with relevant guidelines and regulations. In addition, the adult patient gave his oral and written informed consent.

## Results

### Description of the clinical case

**Figure 2:**
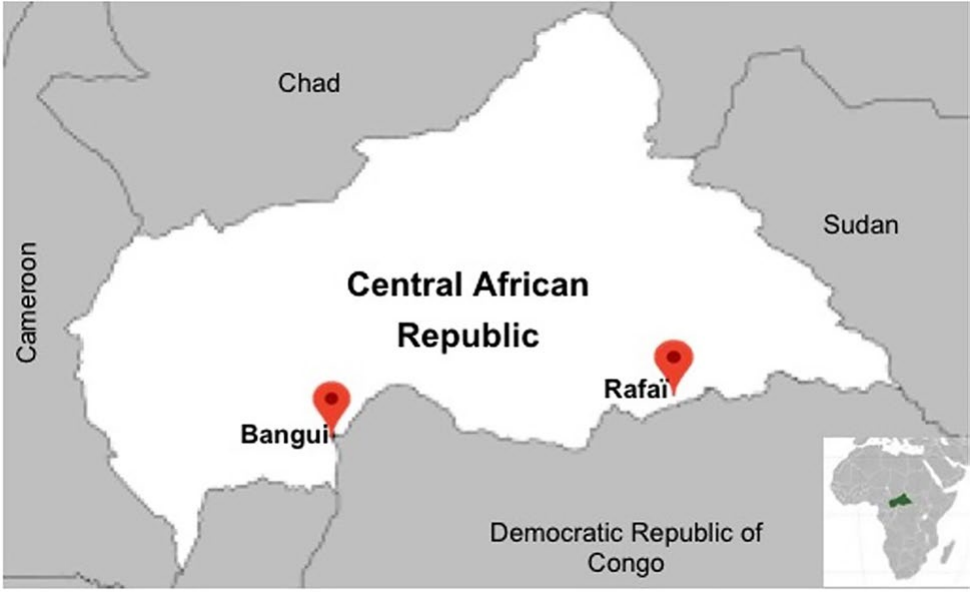
Map of the Central African Republic in Africa. The map was edited in Microsoft PowerPoint (Version 2020).

A 31-year-old street vendor living in the city of Rafai (Mbomou province, CAR) was admitted to a private health center with skin rashes on his face, chest, palms and genital mucosa in April 2018 (Fig. 2). He also had nausea, inguinal adenopathies, chills, difficulty swallowing, mouth ulcers, itchy lesions and myalgia. Following his admission, two women, aged 21 and 22, also developed skin rashes on their face, chest and genital mucosa. Although the infection of the two young women was clearly linked to the sexual relations they had had with the street vendor, epidemiological investigations were not able to determine the origin of infection for the man. Virological qPCR investigations carried out at IPB determined that the cycle threshold (CT) value for the street vendor was 19.7, and 19.8 and 18.3 for the 21- and 22-year-old women, respectively.

### Taxonomic assignment of reads obtained by MinION sequencing

A total of 146,920 raw reads were obtained with sizes ranging from 77 bp to about 68 kb for a median size of 1946 bp. A taxonomic assignment was obtained for 145,068 reads (98.7%) with the Kraken2 classifier, but only 120,187 reads (81.8%) were assigned with the Kaiju protein-level classifier (Table 1). Regardless of the classifier, the vast majority of the reads were assigned as eukaryotes. Kraken2 assigned 93% of the total reads to the *Homo sapiens* host and Kaiju assigned only 82% (63,192/76,922) of the reads to *Homo sapiens* among all the eukaryote-assigned reads (76,922/121,026). Kraken2 and Kaiju assigned 1423 and 36,102 reads respectively to the superkingdom Bacteria, including 97.26% (138/1423) and 66.72% (24,088/36,102) respectively to the phylum Proteobacteria (Table 1). On the other hand, only Kraken2 assigned 6761 reads as belonging to bacterial metagenomes. Finally, the two classifiers Kraken2 and Kaiju made it possible to highlight respectively 2198 and 2322 reads of double-stranded DNA virus sequences with no RNA stage. Of these reads, Kraken2 assigned 2168 (98.6%) and 2111 (96.4%) as belonging respectively to the family *Poxviridae* and the genus *Orthopoxvirus* and Kaiju followed the same pattern, assigning 2195 (94.5%) and 2137 (92.0%) to the same family and genus respectively. Although Kraken2 assigned 99.2% of the reads (2094/2111) to the MPXV species, Kaiju assigned only 51.9% (1110/2137) of the reads to the MPXV species. In addition, 43% (919/2137) and 5.1% (108/2137) of the reads classified in the genus *Orthopoxvirus* by Kaiju were assigned respectively as an unassigned orthopox or another orthopox species such as cowpox (Table 1). Examination of the taxonomic assignments obtained from these two classifiers showed that the total reads assigned to the family of *Poxviridae* was 2223 reads, of which 2140 reads were common to both classifiers. However, some reads were assigned to the family *Poxviridae* by only one classifier. Analyses of the taxonomic classification results showed that 28 reads were assigned by Kraken2 only, while 55 reads were assigned by Kaiju only. In parallel with the use of these classifiers, mapping all 146,920 raw reads using Minimap2 on a reference MPXV sequence (NC_003310) identified 2171 matching reads, representing about 1.47% of the total reads. Finally, the size of these reads varied between 168 bp and 44 kb for the largest reads.

**Table 1:** Summary of the taxonomic assignments of the reads for each of the two classifiers used (Kraken2 and Kaiju).

	Kraken2	Kaiju
	Number of reads	Number of reads
**Eukaryota**		
*Homo sapiens*	134,702	63,192
Other	0	13,730
Total	134,702 (92.86%)	76,922 (64.02%)
**Bacteria**		
Proteobacteria	1384	24,088
Terrabacteria group	23	7555
Chlamydiae	0	1077
Other	0	3382
Total	1407 (0.97%)	36,102 (30.02%)
**Double-stranded DNA virus, no RNA stage**		
Family *Poxviridae*	2168	2195
Other	–	127
Total	2198 (1.51%)	2322 (1.93%)
Unassigned	–	4841 (4.03%)
Bacterial metagenomes	6761 (4.66%)	–
Total number of reads assigned to a taxon	145,068 (100%)	120,187 (100%)

### Comparison of Illumina- and MinION-sequenced genomes

**Figure 3:**
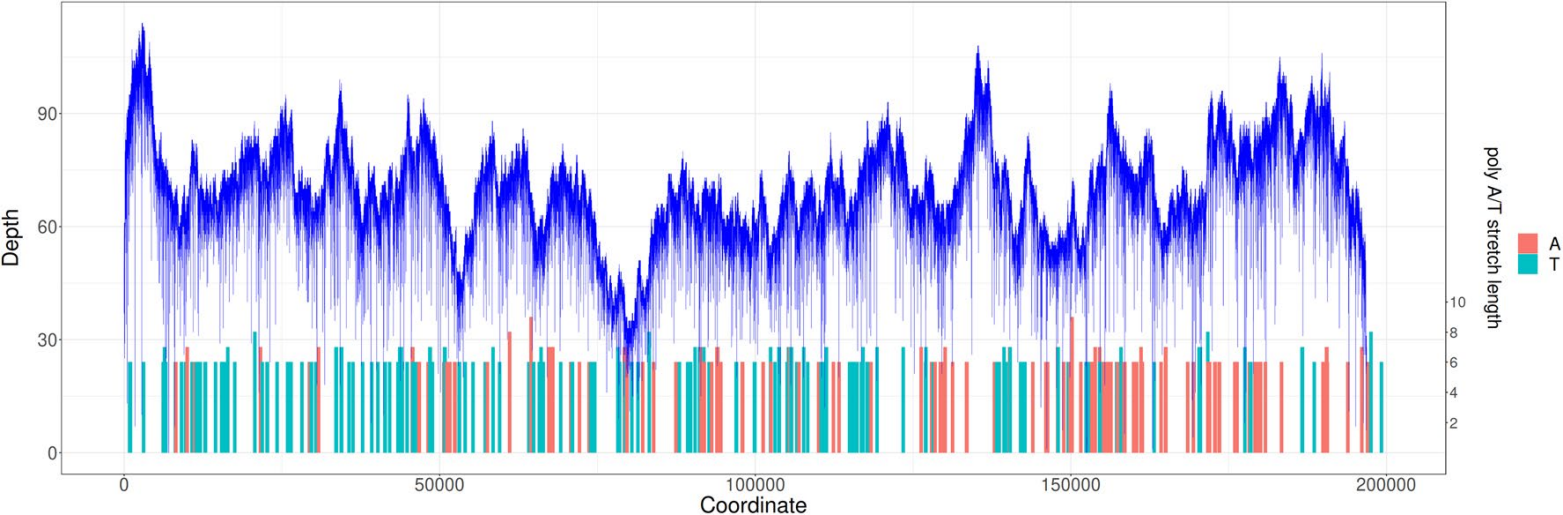
Genome sequencing coverage and location of A/T homopolymers in the monkeypox virus (MPXV) genome. Homopolymer repeat length at each homopolymer position (coordinates) in the genome.

All 2171 MinION reads corresponding to MPXV produced a sequence (MPXV-M) of 196,956 bp with a depth ranging from 12× to 57×. In 94% of the nucleic base positions, the depth was between 30× and 59× with an average depth of 39.72×. Canu was used to both assemble reads classified as belonging to the MonkeyPox genome into draft genomes and then to also remove random sequencing errors. Comparison with the Illumina sequence showed that the two sequences aligned well, but that the MPXV-M sequence was longer than the one initially obtained with the Illumina data (MPXV-I-MN702446), which had a length of only 190,357 bp. The MPXV-I sequence begins at base 573 and ends at base 190,634 with respect to the MPXV-M sequence. However, a retrospective analysis of raw Illumina data found a total of 5924 reads that matched the two ‘missing ends’ of the MPXV-I sequence, even though the majority of these mapped to the terminal repeated regions of both ends of the genome (data not shown). The comparison between the MPXV-I sequence (MN702446) and the MPXV-M sequence revealed insertions or deletions located in homopolymers of at least 6 bases that have been identified in this genome (Table S1 and Fig. 3). However, the major remaining differences between the two genomic sequences were between positions 178,570 and 178,664 with MPXV-M as the reference sequence. Detailed analyses of these differences showed that these 95 bp of the MPXV-I sequence actually corresponded to a region between bases 196,170 and 196,265 of the MPXV-M sequence. However, retrospective analysis using the raw data from Illumina sequencing showed that no reads corresponding to these 95 bp from the region between 178,570 and 178,664 were available. Finally, analysis of the reference sequence (KP849471) that was used for probe design for our targeted enrichment also did not contain this 95 bp region. The absence of this 95 bp region in this reference sequence explains the absence of a capture probe targeted in this genomic region and consequently the absence in our Illumina-sequenced genome (MPXV-I).

**Figure 4:**
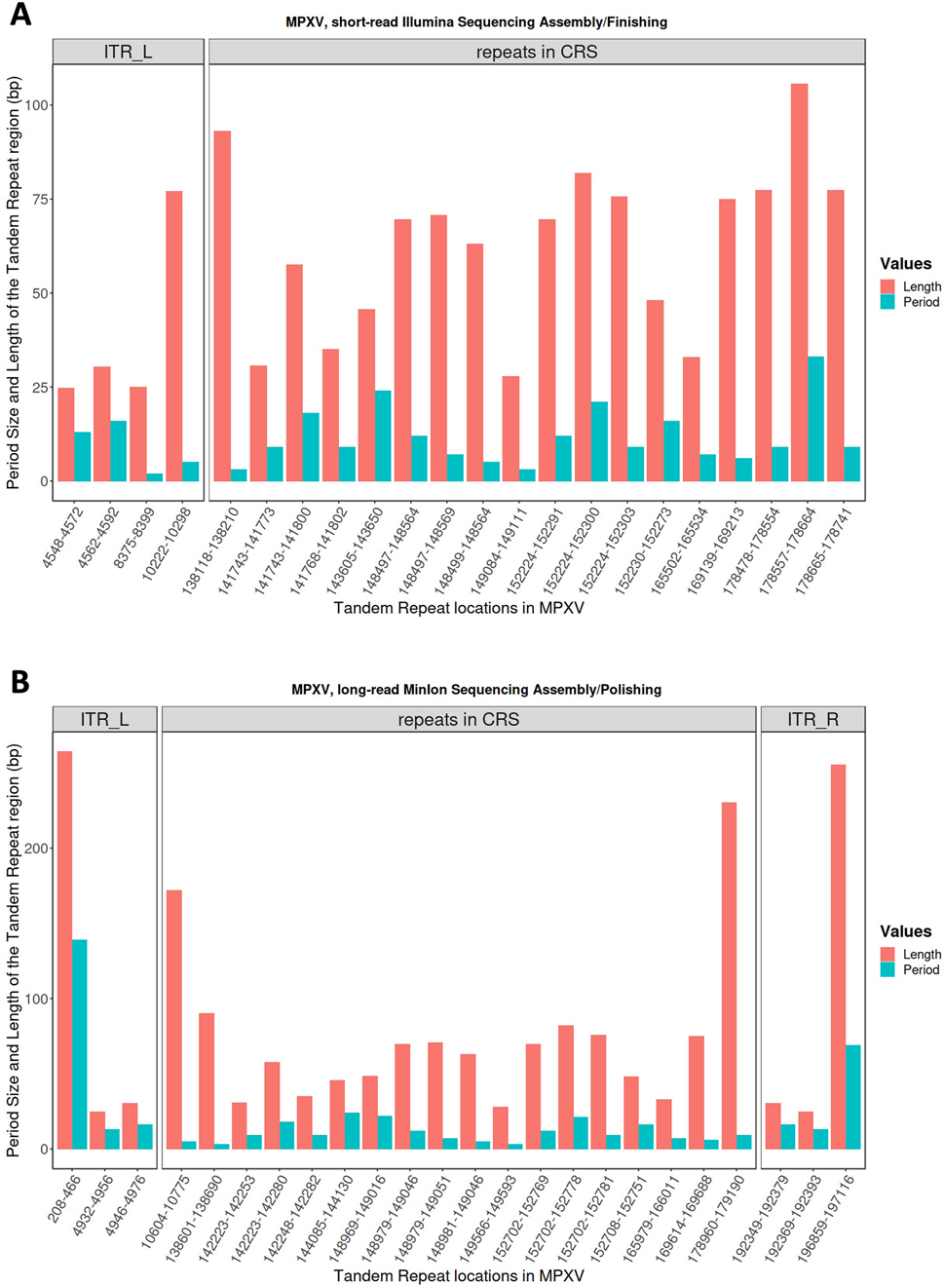
Tandem repeat locations in monkeypox virus genome for short-reads Illumina (**A**) and long-reads MinION (**B**) sequenced genomes using Tander Repeat Finder tool.

In addition to those differences, the central coding region sequence (CRS; roughly between 56,000–120,000) in the MPXV genome is highly conserved and is flanked by variable ends that contain inverted terminal repeats (ITRs). Those ITR’s comprise as much as 1% of the genome, are prone to hair-pin loop-outs ^46,47^ and contain at least 4 ORFs ^48,49^. As such, the ITR regions represent global repeats (i.e., long sequence which is duplicated throughout the genome) and contain local repeated sequences. In the MPXV genome, local tandem repeats are found both within the ITR regions and outside these regions. In contrast to global repeats, the local repeat contains simple sequences, that are tandem duplicated, and therefore pose a special challenge to genome-assembly tools. The GenBank reference MN702446 was obtained using an Illumina short-read sequencing approach. The analysis of this sequence using Tandem Repeat Finder (TRF; ^50^) showed that the left-most ITR sequences could be identified, but showed that the corresponding right-most ITR is missing in the final sequence. Figure 4 shows the tandem repeat locations on the x-axis in the MPXV genome, and the y-axis shows their period size (green bars) and the length (red bars) of the tandem repeat region. This in turn highlights the shortcomings of the assembly of such a repeat-rich sequencing dataset based on short-read sequencing technology. The sequence obtained in the present study, using long-read MinIon technology, analyzed using TRF with the same parameters, showed that the repeats could also be identified in the right-most ITR sequence Fig. 4 shows the equivalent Tandem Repeats found by TRF for the Illumina sequence (Genbank MN702446). The long reads spanning both ITRs could in fact more easily discriminate both ITR’s and allow to finish the genome.

**Figure 5:**
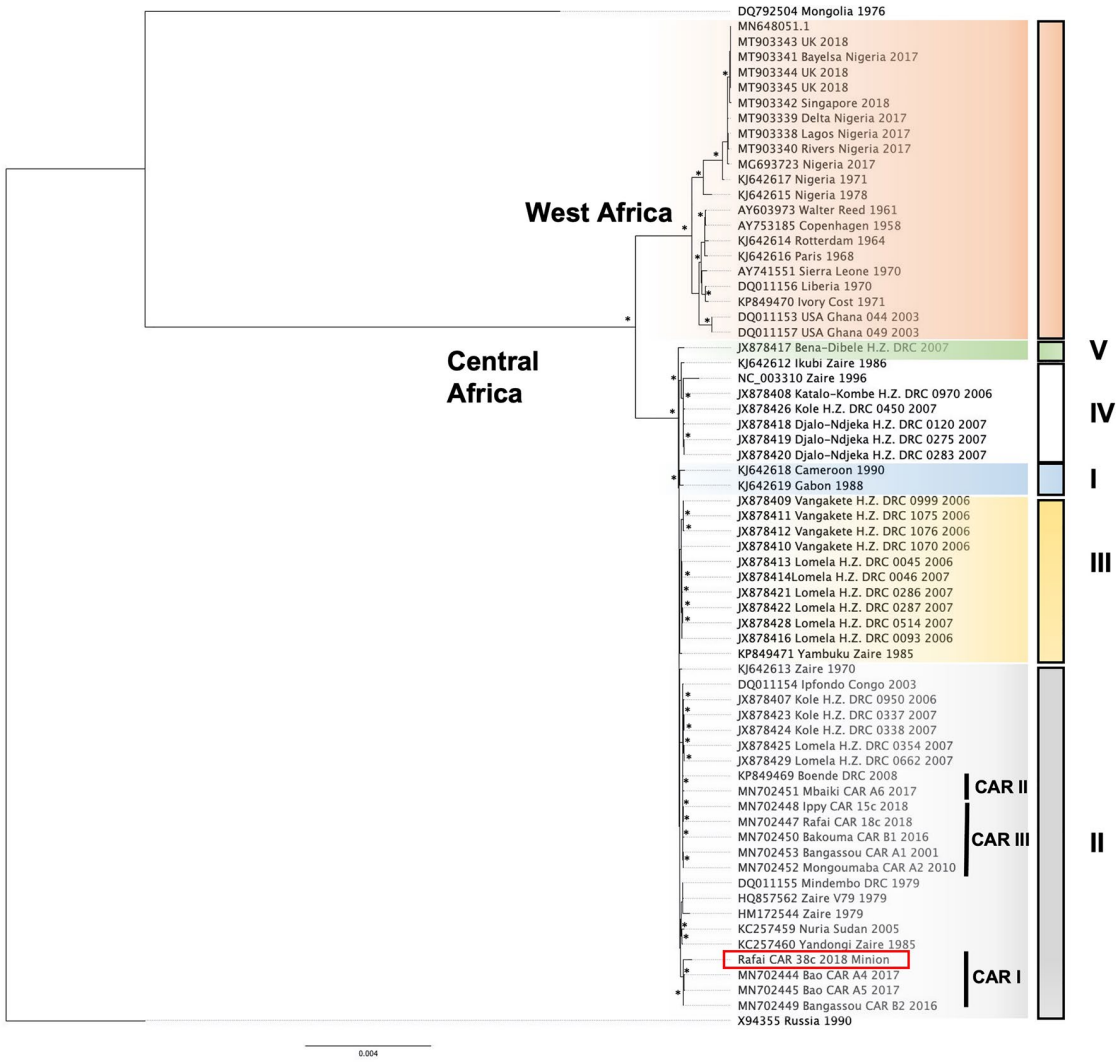
Phylogeny of monkeypox viruses (MPXV) based on complete genomes. The West African clade as well as the 5 groups of the Central African clade were highlighted by different colors: West African clade (orange), Central African group I (blue), II (grey), III (yellow), IV (white) and V (green). The final phylogenetic was generated using FigTree version 1.4.4.

However, despite the presence of these differences in the MPXV-M sequence compared with the MPXV-I sequence, phylogenetic analyses showed that the MPXV-M sequence is positioned in the CAR1 lineage of group II strains belonging to the Central African clade. The position of the MPXV-M sequence, along with the Bao (A4 and A5) and Bangassou (B2) sequences of 2016 and 2017, respectively, is identical to the phylogenetic analyses previously performed in a previous study using all CAR MPXV sequences obtained with Illumina sequencing^19^ (Fig. 5).

### Evaluation of polishing tools on MinION-sequenced genome.

**Figure 6:**
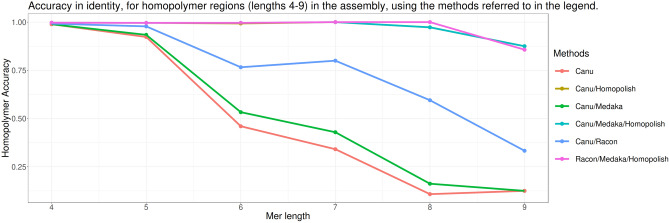
Accuracy of correction of the different polishing tools in combination with Canu for homopolymer sizes between 4 and 9 nt.

**Figure 7:**
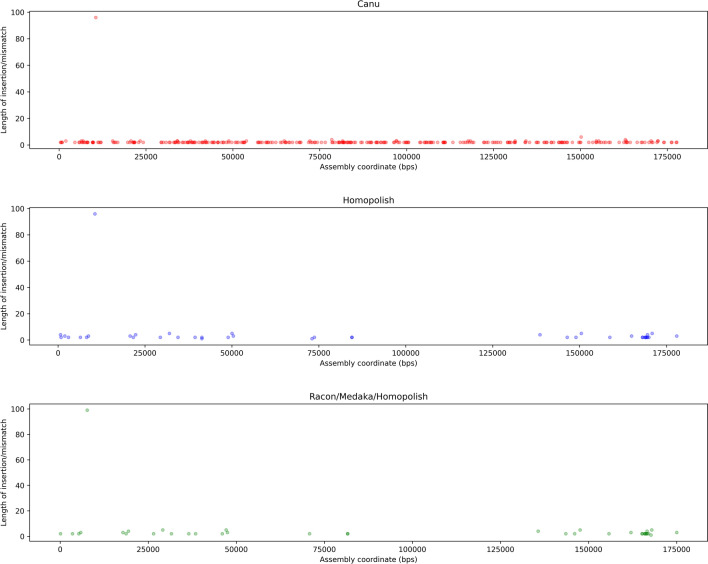
Scatterplot showing effect of polishing using Homopolish (all polymer occurrences).

**Figure 8:**
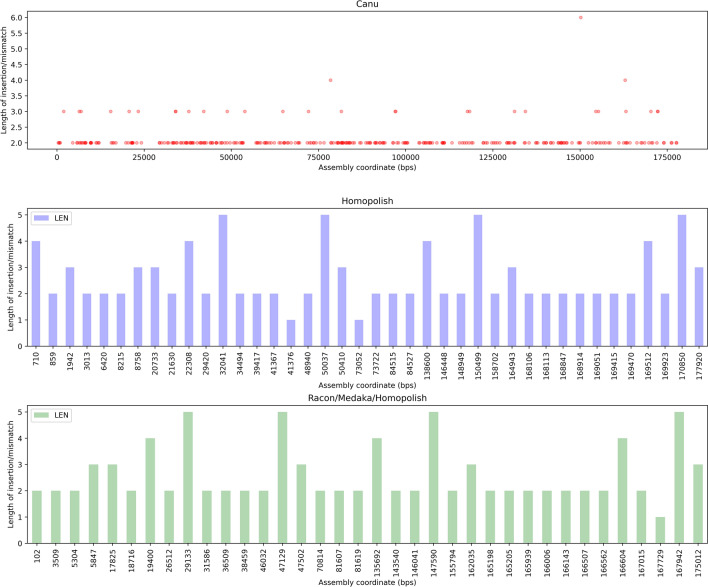
Scatterplot/Barplots showing Effect of polishing using Homopolish (occurrences of polymer of length < 10 bp).

A major issue in nanopore sequencing is the basecalling in homopolymer-rich regions. Basecallers often tend to collapse homopolymers into shorter stretches if the homopolymer length exceeds the number of bases simultaneously influencing the measured current in the nanopore-device, resulting in a higher deletion rate in nanopore reads. As a consequence, the homopolymer containing stretches need to be checked with a dedicated polisher to increase accuracy in downstream steps of the genome finishing process. Therefore, basecalled reads are often assembled into a consensus sequence after which they are mapped back to the assembly to improve the consensus by so-called polishers. Although Canu in fact by default performs a read-correction step, dedicated polishing tools are used in combination with Canu, such as Medaka, Racon or Homopolish, alone or in combination. In order to remove the remaining systematic errors, the efficiency of the polishing operation was evaluated using the sequence for this genome, obtained by Illumina-sequencing, as a reference (MN702446). The different combinations of polishing tools show differences in the number of indels corrected. Indeed, the different combinations of Canu for assembly with polishing tools, such as Canu/Homopolish, but also Canu/Medaka/Homopolish and Canu/Racon/Medaka/Homopolish, give a better correction of indels compared to the other combinations tested. Indeed, the number of indels compared to the use of Canu alone is reduced. It is reduced from 433 to a number varying from 156 to 159 (Table 2). The other combinations tested, such as Canu/Medaka, Canu/Racon or Canu/Racon/Medaka, improve the error correction compared to Canu alone, but less efficiently. Indeed, there are still between 236 and 375 indels depending on the combinations used (Table 2). Even if the assembly performed with Canu is not satisfactory compared to the combinations with the other tools in terms of indels (433 compared to 156 to 375), no mismatch was observed compared to the reference sequence. In contrast, the other combinations, with the exception of Canu/Medaka and Canu/Medaka/Homopolish, introduced 1 or 2 mismatches during the polishing process (Table 2). These mismatches are relatively rare, although they occur in regions of relatively constant sequencing depth (around 70×). The 2 mismatches resulting from polishing with Homopolish, which are 2 A–T transversions, occur in the first half of the genome (about 20–26%, in genome coordinates), while the singular mismatches occurring using Racon, which are A-C transversions, occur in the second identical half of the genome (about 83%, in genome coordinates), and are thus apparently the same SNP events. The combinations tested also have variable efficiency at homopolymeric regions of size 9. Indeed, the Canu/Homopolish combination provides the best accuracy (87.5%) in the polishing procedure in these regions (Fig. 6). Moreover, it is significantly better than the Canu/Medaka combination or Canu/Racon alone (Fig. 6). In contrast, the combination of Medaka with Homopolish did not provide any improvement in error correction in these homopolymeric regions. Furthermore, the genomic coordinates of the homopolymeric sequences appear to be distributed over the entire length of the final assembly after polishing, and are therefore not restricted to the beginning and end of the assembly as illustrated with Figs. 7 and 8, showing all polymeric sequences and aberrations in the length of the deleted polymeric sequences with the precise genomic location of the remaining polymeric sequences. In conclusion, polishing of the raw and corrected reads improved the final assembly, most essentially using the Homopolish tool, notable through the Q-values that are maxing out to 30 Phred value, while increasing only to a small extent the percent identity of the final consensus sequence. The larger number of reads available through the ONT sequencing approach, altogether with the relatively small size of the target genome (200kbp), may contribute to this effect.

**Table 2:** Assembly and Polishing results using Illumina-sequenced genome (MN702446).

Assembly/polishing methods	Q-score	Number of mismatches	Number of InDels
Canu	26.16	0	433
Canu/Homopolish	30.45	2	159
Canu/Medaka	26.78	0	375
Canu/Medaka/Homopolish	30.51	0	159
Canu/Racon	28.71	1	236
Canu/Racon/Medaka	28.39	1	254
Canu/Racon/Medaka/Homopolish	30.5	1	156

## Discussion

In this study, the portable real-time ONT sequencer (MinION) was used for the first time to target and sequence the full MPXV genome from DNA isolated from a human clinical sample in the CAR. Genomic data acquisition and analysis, whether for SGS or TGS, depend on a number of computer-intensive steps. After acquisition, basecalling is the initial step and is ideally performed on a dedicated computer system, given the large amount of data and metadata generated. ONT offers real-time basecalling using cloud services. However, even today, field laboratories in resource-constrained or remote locations often lack stable internet connections, making downstream analyses problematic and severely hindering sample identification in the field. Thus, for field studies, it is necessary to perform private, offline analyses of MinION data. The necessary tools include software for diversity analysis and for classification to rapidly identify species. The most common tools for MinION TGS sequences are carried out using sequence classification tools such as Centrifuge, Kraken2 and Kaiju; reference mapping tools such as MiniMap2 and BWA; and a set of variant analysis tools. Kraken2 was selected among all available DNA-to-DNA classifiers (Kraken2, KrakenUniq, k-SLAM, MegaBLAST, metaOthello, CLARK, CLARK-S, GOTTCHA, TaxMaps, Prophyle, PathSeq, Centrifuge and Karp), because it was available on a dedicated Galaxy platform with an appropriate database (MiniKraken)^35,36,51–60^. In addition, Kraken2 has good performance footprint measures and is very fast on a large number of samples. From the available DNA–protein classifiers (DIAMOND, Kaiju and MMseqs2), Kaiju was chosen because it generally has a much faster classification speed and lower memory footprint requirements than the DIAMOND and MMseqs2 classifiers, without compromising performance^34,61,62^. Most state-of-the-art tools, such as Kraken2 and Kaiju are designed for short reads. They use pseudo alignments, i.e., the exact or approximate matches from reads to a reference as signals to perform classification. Kraken2 utilizes spaced seeds in the storage and querying of minimizers to improve classification accuracy. To run efficiently, Kraken2 requires enough free memory to hold the database (primarily the hash table) in RAM (default database size, 29 GB). The custom extended RefSeq database we used (containing the RefSeq reference libraries for viral, bacterial, fungal, archaea, protozoan and plasmid genomes/proteins, the human GRCh38 human genome/proteins, as well as the NCBI non-redundant nucleotide database containing sequences from large environmental sequencing projects) required a server that had at least 60 GB of RAM, which is currently fairly common. Kraken2 was used in multi-threading mode, and typically required (at the time of writing) approximately 50 min to process a typical sequencing run. For Kaiju, the reference index was built using an equivalent NCBI protein database due to its specifically designed read classification approach, and required approximately 40 GB of RAM. Run times were slightly increased compared with Kraken2, on a comparable number of CPUs. However, given that the two selected classifiers (Kraken2 and Kaiju) operate on different principles, it was almost impossible to compare the accuracy of the classification of each method without using another reference method. Kraken uses k-mers of fixed but variable length identified in the reads. Then, it matches them to the k-mers as defined in its index constructed from reference genomes. Kaiju finds the maximum number of exact matches between the reads and the indexed database. These differences mean that Kaiju should be able to classify more reads, but with less accuracy than Kraken2 ^63^. Likewise, Kaiju classified 36,102 reads as bacteria, whereas Kraken2 classified only 1423 as bacteria. However, the number of reads classified by both methods as belonging to the *Poxviridae* family was similar (2168 versus 2195, respectively, for Kraken2 and Kaiju). Although a few reads were specifically assigned to this family by either method, Kraken2 did not identify any other viral reads, unlike Kaiju, which identified 127. Despite these small differences in the results of viral read classification, both classifiers gave similar results in more than 98% of the cases. Although BLASTN alone would be more efficient compared with either Kraken2 and Kaiju, its use in field conditions is not possible ^34^. Finally, the combination of results obtained with these two classifiers compensated for the lack of precision and the lack of sensitivity by making it possible to identify all the viral reads corresponding to MPXV and to use them for further analysis.

Although this TGS type of technology is known to have a higher error rate compared with SGS on the Illumina platform, few errors were detected in the genome sequence of our MPXV strain. The difference in genome size obtained using the MinION and Illumina sequencing approaches was significant given that the assembly based on the short reads obtained with Illumina was suboptimal in terminal repeat regions. Therefore, the generation of longer reads (with the MinION) provides an advantage in closing the gap on repeat regions such as terminal repeats that are more difficult to assemble with a short-read sequencing approach. In addition, TGS was able to 'correct' a region that had not been correctly sequenced and assembled from the data generated by Illumina sequencing. A study on an old African swine fever virus (ASFV) strain from 2007 resequenced using TGS also improved the length of the inverted terminal repeat sequences compared with the genome obtained previously with SGS (Illumina)^64^. In addition, a major issue in nanopore sequencing is the basecalling in homopolymer-rich regions. Basecaller can accurately call homopolymers up to six bases in length, but without significant improvement for longer homopolymers. Current basecallers often include homopolymer correction settings, although the effect appears to be small, also in our dataset. Therefore, in order to resolve mismatched stretches related to homopolymer sequences, polishers such as Nanopolish and HomoPolish propose to map the reads back to the assembly in order to improve the assembly-consensus, which after polishing is in general very high, but depending on the genome size, it can be a computationally expensive process. As in this study, the 71 detected errors were homopolymers^64^. Polishing the Canu assemblies using NanoPolish (v 0.13.2) is recommended to improve the accuracy of the sequence. Unfortunately, here, polishing did not improve either the final assembly or the accuracy of the final sequence, due to the mapping-based read ordering described above, which was primarily intended to facilitate and guide the assembly process. In contrast, HomoPolish (https://github.com/ythuang0522/homopolish)^65^, a tool relying on a machine-learning model, trained to correct systematic errors that occur in Nanopore sequencing, corrected a number of mismatches and provided a polished version of the assembly compared with the original reference sequence (NC_003310). However, using the reference sequence (obtained with Illumina) did not correct the errors observed in the homopolymers. In addition, using an alternative assembly method based on SPAdes (v. 3.10) to check the robustness of the assembly approach did not produce better assembly results (data not shown).

Despite the differences observed in the homopolymer regions when sequencing the MPXV and ASFV genomes compared with SGS, TGS identifies and characterizes these viral genomes much faster than SGS, and allows to more clearly discriminate ITR sequences, which in turn allows to obtain a longer finished assembly. With SGS, the raw data can only be analyzed after sequencing has been completed, but the MinION data can be analyzed in real time. For example, the ASFV virus was identified as early as 6 min after the start of sequencing and the whole genome within hours^66^. Many examples of the use of TGS have shown that real-time data analysis is the main advantage of this technology over SGS technologies. Rapid identification is a major advantage, especially for viruses that spread very quickly and have serious life-threatening consequences. Although TGS is not the most suitable tool for rapid differential diagnosis of MPXV to distinguish it from Varicella-Zoster virus, its main advantage resides in the very fast identification of the origin of the strain. Our phylogenetic analysis showed that the MPXV-M sequence was sufficiently precise to position it correctly in the CAR1 lineage^19^. Molecular data obtained with TGS makes it possible to identify the relationships between detected cases more rapidly, especially those identified in the same city within a very short timeframe. For instance, in the city of Rafai, several cases had been reported in the space of a few weeks between March and April 2018^67^, but only phylogenetic analyses were able to resolve the specific relationship between them^19^. Similarly, early differential diagnosis of MPXV, distinguishing it from Varicella-Zoster virus, and as close as possible to the index case can help limit its spread to the immediate environment. Secondary cases frequently occur either among family members or among staff at the healthcare facility where the cases are treated. Rapid identification of monkeypox within a few hours can facilitate the isolation of the patient, where possible, and the increased use of personal protective equipment to prevent the spread of the disease. Even though the use of MinION technology is relatively simple and compatible with field use, the handling of biological samples (blood, scabs or pustular lesions, etc.) where a potentially highly contagious agent is present may expose the investigator, without adequate protective equipment, to infection until the sample under investigation is fully inactivated.

In conclusion, this study confirms the usefulness of MinION technology for sequencing the genome of an MPXV virus in the context of an outbreak. Here, we show that the data obtained from directly sequencing DNA extracted from a lesion is sufficient to obtain the complete genome of the virus. The quality of the sequence obtained is suitable to provide information on the origin of the virus with sufficient accuracy despite minor errors related to the acquisition of reads observed in the homopolymeric regions.

## Supplementary Information

Supplementary Table S1.

## Data Availability

The corresponding MinION raw data are available under the BioProject ID (PRJNA762014).

## References

[1] McCollum AM, Damon IK (2014). Human monkeypox. Clin. Infect. Dis..

[2] Jezek Z, Szczeniowski M, Paluku KM, Mutombo M (1987). Human monkeypox: Clinical features of 282 patients. J. Infect. Dis..

[3] Pv M, Andersen E, Petersen K, Birch-Andersen A (1959). A pox-like disease in cynomolgus monkeys. Acta Pathol. Microbiol. Scand..

[4] Khodakevich L, Jezek Z, Kinzanzka K (1986). Isolation of monkeypox virus from wild squirrel infected in nature. Lancet (London, England).

[5] Radonic A (2014). Fatal monkeypox in wild-living sooty mangabey, Cote d'Ivoire, 2012. Emerg. Infect. Dis..

[6] Reynolds MG, Doty JB, McCollum AM, Olson VA, Nakazawa Y (2019). Monkeypox re-emergence in Africa: A call to expand the concept and practice of One Health. Expert Rev. Anti Infect. Ther..

[7] Hutin YJ (2001). Outbreak of human monkeypox, Democratic Republic of Congo, 1996 to 1997. Emerg. Infect. Dis..

[8] Khodakevich L, Jezek Z, Messinger D (1988). Monkeypox virus: Ecology and public health significance. Bull. World Health Organ..

[9] Meyer H (2002). Outbreaks of disease suspected of being due to human monkeypox virus infection in the Democratic Republic of Congo in 2001. J. Clin. Microbiol..

[10] Berthet N (2011). Maculopapular lesions in the Central African Republic. Lancet (London, England).

[11] Nakoune E (2017). A nosocomial outbreak of human monkeypox in the Central African Republic. Open Forum Infect. Dis..

[12] Rimoin AW (2010). Major increase in human monkeypox incidence 30 years after smallpox vaccination campaigns cease in the Democratic Republic of Congo. Proc. Natl. Acad. Sci. USA.

[13] Sklenovska N, Van Ranst M (2018). Emergence of monkeypox as the most important orthopoxvirus infection in humans. Front. Public Health.

[14] Eteng WE (2018). Notes from the field: Responding to an outbreak of monkeypox using the one health approach—Nigeria, 2017–2018. MMWR Morb. Mortal. Wkly Rep..

[15] Faye O (2018). Genomic characterisation of human monkeypox virus in Nigeria. Lancet. Infect. Dis.

[16] Erez N (2019). Diagnosis of imported monkeypox, Israel, 2018. Emerg. Infect. Dis..

[17] Sadeuh-Mba SA (2019). Monkeypox virus phylogenetic similarities between a human case detected in Cameroon in 2018 and the 2017–2018 outbreak in Nigeria. Infect. Genet. Evolut. J. Mol. Epidemiol. Evolut. Genet. Infect. Dis..

[18] Vaughan A (2018). Two cases of monkeypox imported to the United Kingdom, September 2018. Euro Surveill..

[19] Berthet N (2021). Genomic history of human monkey pox infections in the Central African Republic between 2001 and 2018. Sci. Rep..

[20] Gardy J, Loman NJ, Rambaut A (2015). Real-time digital pathogen surveillance—The time is now. Genome Biol..

[21] Pop M, Salzberg SL (2008). Bioinformatics challenges of new sequencing technology. Trends Genet..

[22] Keller MW (2018). Direct RNA sequencing of the coding complete influenza A virus genome. Sci. Rep..

[23] Castro-Wallace SL (2017). Nanopore DNA sequencing and genome assembly on the international space station. Sci. Rep..

[24] Goordial J (2017). In situ field sequencing and life detection in remote (79 degrees 26'N) Canadian high Arctic permafrost ice wedge microbial communities. Front. Microbiol..

[25] Quick J (2016). Real-time, portable genome sequencing for Ebola surveillance. Nature.

[26] Mbala-Kingebeni P (2019). Rapid confirmation of the Zaire Ebola virus in the outbreak of the Equateur Province in the Democratic Republic of Congo: Implications for public health interventions. Clin. Infect. Dis..

[27] Kafetzopoulou LE (2019). Metagenomic sequencing at the epicenter of the Nigeria 2018 Lassa fever outbreak. Science (New York, N.Y.).

[28] Jia L (2020). Nanopore sequencing of African swine fever virus. Sci. China Life Sci..

[29] Cohen-Gihon I (2020). Identification and whole-genome sequencing of a monkeypox virus strain isolated in Israel. Microbiol. Resour. Announc..

[30] Mauldin MR (2020). Exportation of monkeypox virus from the African continent. J. Infect. Dis..

[31] Koren S (2017). Canu: Scalable and accurate long-read assembly via adaptive, javax.xml.bind.JAXBElement@1d401af-mer weighting and repeat separation. Genome Res..

[32] Meyer H, Ropp SL, Esposito JJ (1997). Gene for A-type inclusion body protein is useful for a polymerase chain reaction assay to differentiate orthopoxviruses. J. Virol. Methods.

[33] Panning M, Asper M, Kramme S, Schmitz H, Drosten C (2004). Rapid detection and differentiation of human pathogenic orthopox viruses by a fluorescence resonance energy transfer real-time PCR assay. Clin. Chem..

[34] Menzel P, Ng KL, Krogh A (2016). Fast and sensitive taxonomic classification for metagenomics with Kaiju. Nat. Commun..

[35] Wood DE, Lu J, Langmead B (2019). Improved metagenomic analysis with Kraken 2. Genome Biol..

[36] Wood DE, Salzberg SL (2014). Kraken: Ultrafast metagenomic sequence classification using exact alignments. Genome Biol..

[37] Ondov BD, Bergman NH, Phillippy AM (2011). Interactive metagenomic visualization in a Web browser. BMC Bioinform..

[38] Li H (2018). Minimap2: Pairwise alignment for nucleotide sequences. Bioinformatics (Oxford, England).

[39] Garrison E, Marth G (2012). Haplotype-based variant detection from short-read sequencing. arXiv 1207.3907. Proc. R. Soc. B.

[40] Cingolani P (2012). A program for annotating and predicting the effects of single nucleotide polymorphisms, SnpEff: SNPs in the genome of *Drosophila melanogaster* strain w1118; iso-2; iso-3. Fly.

[41] Li H, Durbin R (2009). Fast and accurate short read alignment with Burrows-Wheeler transform. Bioinformatics (Oxford, England).

[42] Li H (2009). The sequence alignment/map format and SAMtools. Bioinformatics (Oxford, England).

[43] Katoh K, Standley DM (2013). MAFFT multiple sequence alignment software version 7: Improvements in performance and usability. Mol. Biol. Evol..

[44] Nakamura T, Yamada KD, Tomii K, Katoh K (2018). Parallelization of MAFFT for large-scale multiple sequence alignments. Bioinformatics (Oxford, England).

[45] Minh BQ (2020). IQ-TREE 2: New models and efficient methods for phylogenetic inference in the genomic era. Mol. Biol. Evol..

[46] Likos AM (2005). A tale of two clades: monkeypox viruses. J. Gen. Virol..

[47] Tulman ER (2006). Genome of horsepox virus. J. Virol..

[48] Hendrickson RC, Wang C, Hatcher EL, Lefkowitz EJ (2010). Orthopoxvirus genome evolution: the role of gene loss. Viruses.

[49] Shchelkunov SN (2001). Human monkeypox and smallpox viruses: Genomic comparison. FEBS Lett..

[50] Benson G (1999). Tandem repeats finder: A program to analyze DNA sequences. Nucleic Acids Res..

[51] Ainsworth D, Sternberg MJE, Raczy C, Butcher SA (2017). k-SLAM: Accurate and ultra-fast taxonomic classification and gene identification for large metagenomic data sets. Nucleic Acids Res..

[52] Breitwieser FP, Baker DN, Salzberg SL (2018). KrakenUniq: confident and fast metagenomics classification using unique k-mer counts. Genome Biol..

[53] Břinda, K., Salikhov, K., Pignotti, S. & Kucherov, G. ProPhyle 0.3.1.0. *Zenodo* (2017).

[54] Freitas TAK, Li P-E, Scholz MB, Chain PSG (2015). Accurate read-based metagenome characterization using a hierarchical suite of unique signatures. Nucleic Acids Res..

[55] Kim D, Song L, Breitwieser FP, Salzberg SL (2016). Centrifuge: Rapid and sensitive classification of metagenomic sequences. Genome Res..

[56] Kostic AD (2011). PathSeq: Software to identify or discover microbes by deep sequencing of human tissue. Nat. Biotechnol..

[57] Liu X (2018). A novel data structure to support ultra-fast taxonomic classification of metagenomic sequences with k-mer signatures. Bioinformatics (Oxford, England).

[58] Ounit R, Lonardi S (2016). Higher classification sensitivity of short metagenomic reads with CLARK-S. Bioinformatics (Oxford, England).

[59] Ounit R, Wanamaker S, Close TJ, Lonardi S (2015). CLARK: Fast and accurate classification of metagenomic and genomic sequences using discriminative k-mers. BMC Genomics.

[60] Reppell M, Novembre J (2018). Using pseudoalignment and base quality to accurately quantify microbial community composition. PLoS Comput. Biol..

[61] Buchfink B, Xie C, Huson DH (2015). Fast and sensitive protein alignment using DIAMOND. Nat. Methods.

[62] Steinegger M, Söding J (2017). MMseqs2 enables sensitive protein sequence searching for the analysis of massive data sets. Nat. Biotechnol..

[63] Corvelo A, Clarke WE, Robine N, Zody MC (2018). taxMaps: Comprehensive and highly accurate taxonomic classification of short-read data in reasonable time. Genome Res..

[64] Forth JH (2019). A deep-sequencing workflow for the fast and efficient generation of high-quality African swine fever virus whole-genome sequences. Viruses.

[65] Huang YT, Liu PY, Shih PW (2021). Homopolish: A method for the removal of systematic errors in nanopore sequencing by homologous polishing. Genome Biol..

[66] O'Donnell VK (2019). Rapid sequence-based characterization of African swine fever virus by use of the Oxford nanopore MinION sequence sensing device and a companion analysis software tool. J. Clin. Microbiol..

[67] WHO. *Weekly Bulletin on Outbreaks and Other Emergencies, Week 26 2018* (2018). http://apps.who.int/iris/bitstream/handle/10665/272981/OEW26-2329062018.pdf*. *Accessed 09 July 2018 (2018).

