# Supplementary material for: Nanopore sequencing of a monkeypox virus strain isolated from a pustular lesion in the Central African Republic
Source: Sci Rep. 2022 Jun 24;12:10768. doi: 10.1038/s41598-022-15073-1 (PMC9232561; doi:10.1038/s41598-022-15073-1)
Supplement: Supplementary file 1 — Supplementary Table S1. [file 41598_2022_15073_MOESM1_ESM.docx]

**Table S1. Summary of homopolymer characteristics identified in the monkeypox virus genome**

| Number of bases in homopolymer | Total of homopolymers | Number of homopolymers with A | Number of homopolymers with T |
| --- | --- | --- | --- |
| N=6 | **270** | 130 | 140 |
| N=7 | **94** | 35 | 59 |
| N=8 | **37** | 16 | 21 |
| N=9 | **6** | 3 | 3 |
| N=10 | **2** | 0 | 2 |
| N=11 | **1** | 1 | 0 |
